# Autonomous Ground Vehicle Lane-Keeping LPV Model-Based Control: Dual-Rate State Estimation and Comparison of Different Real-Time Control Strategies

**DOI:** 10.3390/s21041531

**Published:** 2021-02-23

**Authors:** Julián M. Salt Ducajú, Julián J. Salt Llobregat, Ángel Cuenca, Masayoshi Tomizuka

**Affiliations:** 1 Department of Automatic Control, LTH, Lund University, 221 00 Lund, Sweden; julian.salt_ducaju@control.lth.se; 2 Instituto Universitario de Automática e Informática Industrial, Universitat Politècnica de València, 46022 València, Spain; acuenca@isa.upv.es; 3 Mechanical Engineering Department, University of California, Berkeley, CA 94720, USA; tomizuka@berkeley.edu

**Keywords:** autonomous vehicle, dual-rate control, dual-rate EKF, MPC, LPV model

## Abstract

In this contribution, we suggest two proposals to achieve fast, real-time lane-keeping control for Autonomous Ground Vehicles (AGVs). The goal of lane-keeping is to orient and keep the vehicle within a given reference path using the front wheel steering angle as the control action for a specific longitudinal velocity. While nonlinear models can describe the lateral dynamics of the vehicle in an accurate manner, they might lead to difficulties when computing some control laws such as Model Predictive Control (MPC) in real time. Therefore, our first proposal is to use a Linear Parameter Varying (LPV) model to describe the AGV’s lateral dynamics, as a trade-off between computational complexity and model accuracy. Additionally, AGV sensors typically work at different measurement acquisition frequencies so that Kalman Filters (KFs) are usually needed for sensor fusion. Our second proposal is to use a Dual-Rate Extended Kalman Filter (DREFKF) to alleviate the cost of updating the internal state of the filter. To check the validity of our proposals, an LPV model-based control strategy is compared in simulations over a circuit path to another reduced computational complexity control strategy, the Inverse Kinematic Bicycle model (IKIBI), in the presence of process and measurement Gaussian noise. The LPV-MPC controller is shown to provide a more accurate lane-keeping behavior than an IKIBI control strategy. Finally, it is seen that Dual-Rate Extended Kalman Filters (DREKFs) constitute an interesting tool for providing fast vehicle state estimation in an AGV lane-keeping application.

## 1. Introduction

Self-driving cars have been increasing in popularity year after year. They are the type of Autonomous Ground Vehicle (AGV) that has received the greatest share of attention, both in academia and in industry, because of the possibility that they can shift the paradigm of transportation systems. An essential concern in the development of these automated driving systems is the ability to obtain a controller that is able to make the vehicle follow a pre-established path. This problem is often considered, in a hierarchical manner, as the low level control of the AGV in opposition to a high level control, which is focused on path or trajectory generation based on the awareness of the environment that surrounds the vehicle.

The lateral vehicle control takes care of the path-tracking problem. The path is composed of a sequence of positions and orientations in the plane, and the controller has to make sure that the vehicle follows them. In order to control the vehicle, two input variables are often considered: the steering angle (which is modified by acting on the steering wheel) and the longitudinal acceleration (modified via throttle). If the vehicle must follow a feasible collision-free pre-computed path with no time constraints, the problem is known as lane-keeping. On the contrary, if each pair of the position and orientation of the pre-defined path has a time stamp associated with them, the problem is considered as a trajectory-tracking problem. Since time constraints are not considered in this application, the steering angle can be chosen as the only control variable, disregarding the longitudinal acceleration.

To achieve successful lateral vehicle control, it is necessary that a set of sensors (GPS, IMU, and others) is embedded in the vehicle. These sensors measure some variables such as position, velocity, acceleration, orientation, and change of orientation, at different rates. The use of the celebrated Kalman filter [1,2,3] enables fusing all of them with the aim of being conveniently utilized by the control stage. Additionally, several authors have proposed different models to describe the lateral dynamics of the vehicle. The use of a kinematic bicycle model has been widely extended, where each axle is considered as a single wheel [4]. The model was later expanded through a dynamic expression that links, among other variables, the inertial heading time evolution with the steering wheel angle. The lateral dynamics of wheeled ground vehicles is determined by the highly nonlinear forces occurring in their tires. For this reason, most of the models that have been suggested [4,5,6,7,8,9,10] are nonlinear models.

Therefore, in order to use the Kalman filter in a proper way, it needs to be formulated via its extended or unscented versions (see, e.g., [11,12]). In the present approach, the Extended Kalman Filter (EKF) was chosen, not only for fusing all the data provided by the different sensing devices, but also for estimating the nonlinear behavior of the vehicle’s dynamics, providing unavailable (not measurable) variables if needed and reducing the possible process and measurement noise effect. Since every sensor may work at a different rate (the GPS and velocimeter usually work at slower rates, but the IMU works at a faster rate), which may be slower than the actuation (control) rate, a multi-rate EKF may be needed. In our proposal, the different output variables are assumed to be sensed at different rates, but the internal state of the filter is only updated at a rate *M*-times slower than the actuation one. This leads to a Dual-Rate EKF (DREKF).

The literature on the DREKF is scarce and scattered. Some works appear in biomedicine, concretely in the field of electrocardiogram signal denoising, where the DREKF has been used in order to better estimate system states that are not updated in all time instances and avoid unwanted errors in the estimation procedure [13,14]. Unmanned Aerial Vehicles (UAVs) are another field where the DREKF has been employed with the aim of estimating state variables from few measurements, which come from a low cost, low rate GPS [15].In robotics, although factor-graph-based methods (GT-SAM, OkVIS, Cartographer, etc.) are prevalent, the DREKF can also be utilized for ego-motion estimation so as to fuse low-rate vision and fast-rate inertial measurements in the context of the simultaneous localization and mapping problem [16,17]. To the best of the authors’ knowledge, the DREKF has never been explicitly formulated in the AGV’s framework.

Several authors have explored the topic of motion planning and control for AGVs (see, e.g., [18]) using different control approaches such as Linear Quadratic Regulator (LQR) control, inverse kinematics controller, Model Predictive Control (MPC), and some attempts with classical control (PID, lead-lag) [4,19]. In particular, MPC has been widely used in trajectory reference tracking for self-driving cars [8,20,21,22,23,24,25], since it enables calculating and optimizing the sequence of future control inputs by using an explicit model [26].

Depending on the control scheme selected, choosing a nonlinear model can cause a relevant increase in the calculation time, which may endanger the feasible real-time solving of the controller. On the contrary, a Linear Time-Invariant (LTI) model might be insufficient to describe the vehicle’s dynamics, especially if high lateral tire forces are involved [6,9,10]. Linear Parameter Varying (LPV) models have been regarded as a trade-off between model accuracy and computational complexity [24,25,27,28,29,30].

Previous contributions on LPV-MPC for reference tracking in ground vehicles have shown promising results [24,25]. Here, the nonlinearities of the vehicle’s dynamics are embedded into the model’s varying parameters, which may cause prediction errors for long time horizons if the variation from the operating point is meaningful throughout this time interval. In a recent contribution [31], a learning algorithm for vehicular dynamics [32] was applied to an LPV car dynamics’ model [27], to optimize the prediction results over a long prediction horizon. It would be interesting to analyze its behavior in a realistic scenario when used in model-based control.

Moreover, as previously mentioned, in this control problem, some sensors work at a slower rate. In order to reach a good control performance, this rate may not be appropriate to update the controller output. Then, instead of using a DREKF to provide a single, fast-rate controller with faster estimates, a dual-rate controller may be considered to generate faster control actions from slower measurements.

The main contributions of this article are three. First, were present an LPV-MPC design that considers a model identified specifically for longer time scale predictions such as the ones handled by MPC. Second, we introduce a Dual-Rate EKF (DREKF) that allows a fast state update using, but not limited to, slow and noisy measurements in a autonomous vehicle control context. Third, we compare and analyze two different low computational complexity, dual-rate approaches for lateral vehicle controlling in the presence of process and measurement Gaussian noise. The first of the two approaches considered in the third contribution uses a single, fast-rate feedforward controller, which is designed from an Inverse Kinematics Bicycle (IKIBI) model. The second considers an MPC controller, which can be designed from a new LPV optimized model and with a prediction horizon that allows generating a fast-rate control signal from the slow-rate measurements.

The paper is organized as follows. Section 2 details the design aspects for each control approach (IKIBI and MPC). Then, the DREKF is introduced in Section 3. Simulated experiments are introduced and justified in Section 4, and their results are presented and discussed in Section 5. Finally, some conclusions summarize the present work in Section 6.

## 2. Control Strategies

There are diverse control laws devoted to vehicle lane-keeping, commonly called steering controllers. In this section, two widely used methods with some variations are considered: the Inverse Kinematic Bicycle model (IKIBI) and the Linear Parameter Varying-Model Predictive Control (LPV-MPC).

**Figure 1:**
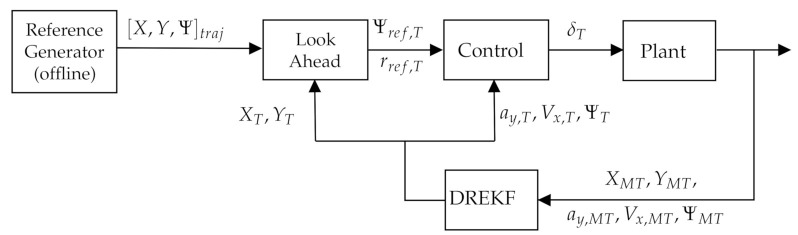
Proposed closed-loop control. DREKF, Dual-Rate Extended Kalman Filter.

In both cases, the purpose is to use the steering front wheels’ angle δ as the control action in order to follow the desired path. The complete path, ${\left[\begin{array}{ccc} X & Y & \Psi \end{array}\right]}_{traj}$, is planned offline, and depending on the controller election, the next yaw rate, $r_{ref}$, or yaw position goal, $\Psi_{ref}$, is delivered by a pure pursuit procedure with a coherent look-ahead distance *L* [7,19,33,34]. Figure 1 shows a schematic view of this process. The Dual-Rate Extended Kalman Filter (DREKF) proposed for state estimation can also be seen in Figure 1 and is further explained in Section 3.

### 2.1. Inverse Kinematic Bicycle Model-Based Controller

In this work, the IKIBI is used by adding a proportional feedforward controller in order to consider the yaw rate measurement *r*. The control law yields:

(1) $$\delta \left(k+1\right)=\left[atan_{2}\left(\frac{r_{ref}L_{v}}{v_{x}\left(k\right)}+K_{p}\left(r_{ref}\left(k+1\right)-r\left(k\right)\right)\right)\right]\gamma$$

where $K_{p}$ is the feedforward controller’s proportional gain, $r_{ref}$ is the yaw rate goal established by the pure pursuit, $L_{v}$ is the vehicle’s longitudinal length, and γ is a vehicle coefficient that translates the tire angle into the steering angle. Since the input signal is considered directly as the tire angle, γ=1. Furthermore, the function $atan_{2}$ represents the fourth-quadrant inverse tangent.

### 2.2. Linear Parameter Varying-Model Predictive Control

Model predictive control can be used for lateral vehicle control [8,20,21]. A linear model of the system should be considered to implement an MPC controller in real time in order to avoid computational delays [21,35,36]. The lateral dynamics’ model that is used for this controller was presented in [27]:

(2) $$\dot{\psi }\left(k\right)=\frac{b_{0}+b_{1}z^{-1}+b_{2}z^{-2}}{1+a_{1}z^{-1}+a_{2}z^{-2}}\delta \left(k\right)$$

where $\dot{\psi }$ is the yaw rate in the body frame coordinates of the vehicle and δ the front steering angle. The presented model is Linear Parameter Varying (LPV), and its coefficients ($b_{0-2}$ and $a_{1,2}$) depend on the lateral acceleration and longitudinal velocity in the local body frame of the vehicle. Previous research has suggested an optimized method for identifying the system’s parameters [31] by minimizing the model prediction errors over a long time horizon.

Since the goal of this controller is to follow a trajectory reference described in terms of position (*X* and *Y*) and orientation (Ψ) in absolute coordinates, it is interesting to set the orientation of the vehicle (ψ) as the output of the system rather than its rate of change ($\dot{\psi }$). A forward Euler method is used where:

(3) $$\psi \left(k\right)=\frac{T}{z-1}\dot{\psi }\left(k\right)=\frac{Tz^{-1}}{1-z^{-1}}\dot{\psi }\left(k\right)$$

where T is the sampling period of the system. Then:

(4) $$\psi \left(k\right)=\frac{Tb_{0}z^{-1}+Tb_{1}z^{-2}+Tb_{2}z^{-3}}{1+\left(a_{1}-1\right)z^{-1}+\left(a_{2}-a_{1}\right)z^{-2}-a_{2}z^{-3}}\delta \left(k\right)$$

Therefore, the model used in MPC is:

(5) $$\begin{array}{ccc} x(k+1) & = & Ax(k)+Bu(k) \end{array}$$

(6) $$\begin{array}{ccc} y(k) & = & Cx(k)+Du(k) \end{array}$$

where *u* and *y* are the discrete-time input (δ(k)) and output (ψ(k)) variables, respectively, and:

(7) $$\begin{array}{ccc} A & = & \left[\begin{array}{ccc} 1-a_{1} & a_{1}-a_{2} & a_{2}\\ 1 & 0 & 0\\ 0 & 1 & 0 \end{array}\right] \end{array}$$

(8) $$\begin{array}{ccc} B & = & {\left[\begin{array}{ccc} 1 & 0 & 0 \end{array}\right]}^{T} \end{array}$$

(9) $$\begin{array}{ccc} C & = & \left[\begin{array}{ccc} Tb_{0} & Tb_{1} & Tb_{2} \end{array}\right] \end{array}$$

(10) $$\begin{array}{ccc} D & = & 0 \end{array}$$

The quadratic cost function chosen for solving this problem at each time step *k* is:

(11) $$J_{U}\left(k\right)=\sum_{i=k+1}^{k+M}{\left(y\left(k\right)-y_{ref}\left(k\right)\right)}^{T}Q\left(y\left(k\right)-y_{ref}\left(k\right)\right)+\sum_{i=k}^{k+M-1}u{\left(k\right)}^{T}Ru\left(k\right)$$

where *U* is the input signal sequence of the control horizon that minimizes the cost function over the MPC prediction horizon for every metaperiod and *Q* and *R* are positive semi-definite weight matrices that penalize the controlled variables and inputs, respectively. Furthermore, $y_{ref}\left(k\right)$ is determined by the look-ahead algorithm.

This optimization problem is subject to the discrete-time model of the system (5), (6) and a set of linear constraints on the control and output that preserve the physical feasibility of the solution:

(12) $$\begin{array}{ccc} FU(k) & \leq & f \end{array}$$

(13) $$\begin{array}{ccc} GY(k) & \leq & g \end{array}$$

where *Y* is the sequence of discrete-time output variables in the prediction horizon of the MPC problem. Furthermore, the rate of change of the output variable is limited by the slew rate, *S*, to avoid abrupt vehicle turns that would have a detrimental effect on the passengers’ comfort:

(14) $$l_{\infty }\left(y\left(k+1\right)-y\left(k\right)\right)\leq S$$

The choice of the cost function as convex, as well as a linear model, and convex constraint sets makes the whole problem convex, which is beneficial for the computation of the problem since if a solution exists, it is the globally optimal one [37].

Finally, one of the aspects of MPC is the generation of the sequence of the *M* future discrete-time control actions to achieve the goal reference. However, it is typical that only the first control action is injected. Therefore, MPC is a natural dual-rate control in the sense that it calculates *M* future control actions with each of the measurement data.

## 3. Dual-Rate Extended Kalman Filter

In previous work [31], the set of hardware available (an inertial measurement unit, a differential GPS, and a computer) for data acquisition in the car was able to measure *X*, *Y*, Ψ, and $V_{x}$, but $V_{y}$ and *r* were difficult to access.

Moreover, the measurements are available at different frequencies, the GPS’s at a slow frequency (about 10 Hz) and the same for velocity acquisition. The orientation ψ is acquired by the IMU with a frequency of 100 Hz. For this reason, dual-rate control is a natural proposal to deal with this problem, assuming slow-rate measurements, but a fast (*M*-times faster) steering control action. The acceleration could be varied at a slow frequency. In the case of the dual-rate EKF, it may be needed when some of the measurements are not available due to its slow-rate acquisition (for instance, *X* and *Y* from the GPS). Then, state estimation is carried out at a faster rate from ($V_{x}$, ψ).

Analogously, the DREKF includes a linearization procedure, which is based on the use of the Jacobian matrix (a matrix of partial derivatives). At each time step, this matrix is evaluated with the current predicted states. The DREKF, different from a standard EKF, carries out some slow-rate computations (such as the correction stage) only when output variables are available, that is when they are sensed. Otherwise, predictions are shifted to the next iteration.

The DREKF presented in this section takes a nonlinear model based on Newton’s second law that uses the bicycle model and assumes a constant tire load [5,24,29] for state estimation. Expressing the model in discrete time for period *T* yields:

(15) $$\begin{array}{ccc} F_{yf}\left(k\right) & = & -C_{\alpha f}\arctan\left(\frac{V_{y}\left(k-1\right)+r\left(k-1\right)a}{\max(V_{x}\left(k-1\right),V_{\min})}-\delta \left(k-1\right)\right) \end{array}$$

(16) $$\begin{array}{ccc} F_{yr}\left(k\right) & = & -C_{\alpha r}\arctan\left(\frac{V_{y}\left(k-1\right)-r\left(k-1\right)b}{\max(V_{x}\left(k-1\right),V_{\min})}\right) \end{array}$$

(17) $$\begin{array}{ccc} a_{x}\left(k\right) & = & a_{x}\left(k-1\right) \end{array}$$

(18) $$\begin{array}{ccc} a_{y}\left(k\right) & = & -V_{x}\left(k-1\right)r\left(k-1\right)+\frac{F_{yf}\left(k-1\right)+F_{yr}\left(k-1\right)}{m} \end{array}$$

(19) $$\begin{array}{ccc} \dot{r}\left(k\right) & = & \frac{aF_{yf}\left(k-1\right)\cos\left(\delta \left(k-1\right)\right)-bF_{yr}}{I_{zz}} \end{array}$$

(20) $$\begin{array}{ccc} V_{x}\left(k\right) & = & V_{x}\left(k-1\right)+T\cdot a_{x}\left(k-1\right) \end{array}$$

(21) $$\begin{array}{ccc} V_{y}\left(k\right) & = & V_{y}\left(k-1\right)+T\cdot a_{y}\left(k-1\right) \end{array}$$

(22) $$\begin{array}{ccc} r(k) & = & r\left(k-1\right)+T\cdot \dot{r}\left(k-1\right) \end{array}$$

(23) $$\begin{array}{ccc} X(k) & = & X(k-1)+\\ & + & T\left[V_{x}\left(k-1\right)\cos\left(\psi \left(k-1\right)\right)-V_{y}\left(k-1\right)\sin\left(\psi \left(k-1\right)\right)\right] \end{array}$$

(24) $$\begin{array}{ccc} Y(k) & = & Y(k-1)+\\ & + & T\left[V_{x}\left(k-1\right)\sin\left(\psi \left(k-1\right)\right)+V_{y}\left(k-1\right)\cos\left(\psi \left(k-1\right)\right)\right] \end{array}$$

(25) $$\begin{array}{ccc} \psi (k) & = & \psi (k-1)+T\cdot r(k-1) \end{array}$$

where, as mentioned earlier, *X* and *Y* are the position coordinates in the absolute inertial frame, Ψ is the orientation coordinate also in absolute coordinates, ψ is the orientation coordinate in the body-frame coordinates, *r* its rate of change, and $V_{x}$, $V_{y}$, $a_{x}$, and $a_{y}$ are the velocities and accelerations, respectively, in the body-frame coordinates. Moreover, the different constants that appear in these equations are defined in Appendix A, where their numerical values are also given to allow the reproducibility of the results. Additionally, let us denote this global nonlinear dynamic model as the next state-space representation

(26) $$\begin{array}{c} \left\{\begin{array}{c} \xi \left(k\right)=f\left(\xi \left(k-1\right),n_{1}\left(k-1\right),u\left(k-1\right)\right)\\ z\left(k\right)=h\left(\xi \left(k\right),n_{2}\left(k\right)\right) \end{array}\right. \end{array}$$

where the AGV state ξ(k) is composed of ${\left(V_{x}\left(k\right),V_{y}\left(k\right),X\left(k\right),Y\left(k\right),\psi \left(k\right),r\left(k\right)\right)}^{T}$, the control signal is $u\left(k-1\right)={\left(a_{x}\left(k-1\right),\delta \left(k-1\right)\right)}^{T}$, the output consists of $z\left(k\right)=(V_{x}\left(k\right),X\left(k\right),$${Y\left(k\right),\psi \left(k\right))}^{T}$, and $n_{1}\left(k-1\right)$ and $n_{2}\left(k\right)$ are process and measurement noises, respectively, which are both assumed to be zero mean multivariate Gaussian noises with variance $\bar{Q}\left(k\right)=0.01$ and $\bar{R}\left(k\right)=0.01$, respectively.

Assuming that the notation $\hat{\xi }\left(j|i\right)$ means the state estimated for the instant jT at the instant iT, the prediction and correction steps of the DREKF are defined as follows:

- Fast-rate calculations:
  – Prediction of the next state $\hat{\xi }\left(k|k-1\right)$ and propagation of the covariance P(k|k−1). These computations are calculated ∀k:
    (27) $$\begin{array}{cc} \hat{\xi }\left(k|k-1\right) & =f\left(\hat{\xi }\left(k-1|k-1\right),n_{1}\left(k-1\right),u\left(k-1\right)\right)\\ P(k|k-1) & =A\left(k\right)P\left(k-1|k-1\right)A{\left(k\right)}^{T}+L\left(k\right)\bar{Q}\left(k-1\right)L{\left(k\right)}^{T} \end{array}$$
    where $\hat{\xi }\left(0\right)=E\left[\xi \left(0\right)\right]$ $P\left(0\right)=E\left[\left(\xi (0)-E[\xi (0)]\right){\left(\xi (0)-E[\xi (0)]\right)}^{T}\right]$, E[·] being the expectation, and where A(k) and L(k) are Jacobian matrices computed in order to respectively linearize the process model about the current state and about the process noise:
    (28) $$\begin{array}{cc} \bar{A}\left(k\right) & ={\left.\frac{\partial f}{\partial \xi }\right|}_{\hat{\xi }\left(k-1|k-1\right),n_{1}\left(k-1\right),u\left(k-1\right)}\\ L(k) & ={\left.\frac{\partial f}{\partial n_{1}}\right|}_{\hat{\xi }\left(k-1|k-1\right),n_{1}\left(k-1\right),u\left(k-1\right)} \end{array}$$
  – State and covariance shifts, for $\hat{\xi }\left(k|k\right)$ and P(k|k), respectively. These computations are calculated when measurements are not provided, that is for k≠MT:
    (29) $$\begin{array}{cc} \hat{\xi }\left(k|k\right) & =\hat{\xi }\left(k|k-1\right)\\ P(k|k) & =P(k|k-1) \end{array}$$
- Slow-rate calculations, which are computed when measurements are provided, that is for k=MT:
  – Prediction of the future output $\hat{z}\left(k\right)$:
    (30) $$\hat{z}\left(k\right)=h\left(\hat{\xi }\left(k|k-1\right),n_{2}\left(k\right)\right)$$
  – Computation of the Kalman filter gain K(k):
    (31) $$K\left(k\right)=P\left(k|k-1\right)H{\left(k\right)}^{T}{\left(H\left(k\right)P\left(k|k-1\right)H{\left(k\right)}^{T}+M\left(k\right)\bar{R}\left(k\right)M{\left(k\right)}^{T}\right)}^{-1}$$
    where H(k) and M(k) are Jacobian matrices calculated in order to respectively linearize the output model about the predicted next state and about the measurement noise:
    (32) $$\begin{array}{cc} H(k) & ={\left.\frac{\partial h}{\partial \xi }\right|}_{\hat{\xi }\left(k|k-1\right),n_{2}\left(k\right)}\\ M(k) & ={\left.\frac{\partial h}{\partial n_{2}}\right|}_{\hat{\xi }\left(k|k-1\right),n_{2}\left(k\right)} \end{array}$$
  – Correction of the state $\hat{\xi }\left(k|k\right)$ and correction of the covariance P(k|k):
    (33) $$\begin{array}{cc} \hat{\xi }\left(k|k\right) & =\hat{\xi }\left(k|k-1\right)+K\left(k\right)\left(z\left(k\right)-\hat{z}\left(k\right)\right)\\ P(k|k) & =K\left(k\right)\bar{R}\left(k\right)K{\left(k\right)}^{T}+\left(I-K\left(k\right)H\left(k\right)\right)P\left(k|k-1\right){\left(I-K\left(k\right)H\left(k\right)\right)}^{T} \end{array}$$

Finally, it should be mentioned that if M=1 is assumed, the state and covariance shifts in (29) are replaced by the corrections in (33), resulting in a (single-rate) EKF.

## 4. Implementation

In this section, we present the experiments that were performed in order to compare the two proposed controllers for lane-keeping and justify the appeal of using a DREKF in this application.

The design choices for the controllers and some simulation details are presented first, followed by a discussion of the tests’ selection. Afterwards, we introduce the cost indexes that quantify each controllers’ performance, and we present the results obtained.

### 4.1. Simulation Details and Design Choices for the Controllers

Simulations were carried out using the vehicle parameters of a 2017 Lincoln MKZ on a circuit path. The sampling period of the simulated discrete-time plant was assumed to be T=0.01 s, which is the same as the fastest acquisition frequency of sensors installed in the test-bed vehicle.

The IKIBI-based controller design resulted in $K_{p}=0.55$, and as mentioned earlier, γ=1. On the other hand, the LPV model parameters used for the MPC strategy were obtained from previous research results [31]. Moreover, the convex optimization problem of the MPC was solved using CVXGEN [38].

Moreover, the prediction and the control horizons in the MPC problem were chosen to be equal to 10 steps (N=10), to ensure a small computation time, and the weighting matrices that penalize the output deviation from its reference and the input were, Q=1 and R=0.001, respectively. Since the main goal of this implementation was to obtain an accurate control, the vehicle’s orientation error was penalized much more heavily compared to the input signal.

**Figure 2:**
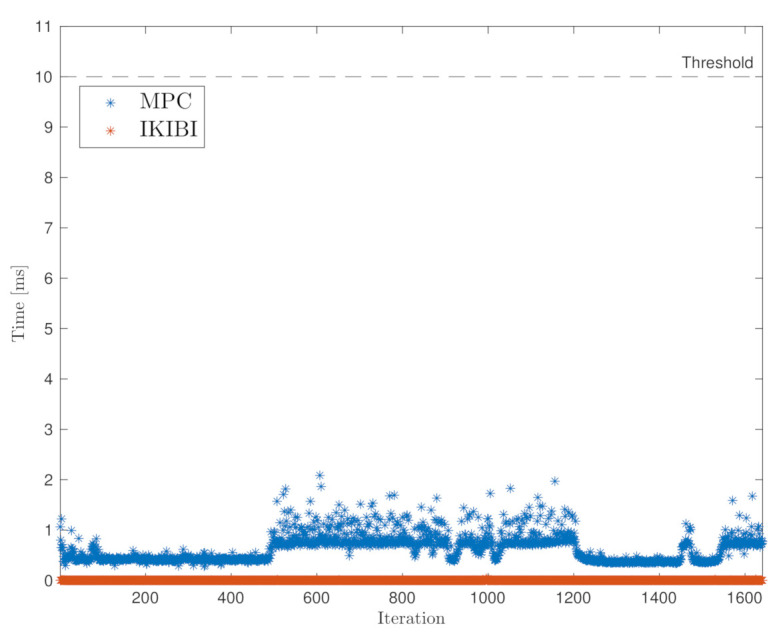
Calculation duration throughout the trajectory.

Furthermore, to verify that the low computational complexity of the two controllers allows a real-time implementation, the calculation time for each controller was computed throughout the entire trajectory. The results are shown in Figure 2. It can be seen that all calculations were below the vehicle’s sampling period, which was equal to 10 milliseconds. The average and standard deviation for the IKIBI and the MPC controllers were equal to 0.003 ± 0.001 milliseconds and 0.637 ± 0.276 milliseconds, respectively. Furthermore, the slowest execution time recorded was 0.0040 milliseconds for the IKIBI controller and 2.086 milliseconds for the MPC controller.

### 4.2. Performed Tests’ Selection

The performed tests compared the behavior of the two proposed controllers, the IKIBI-based controller and MPC-based controller. The tests were performed focusing on the lateral dynamics of the vehicle, and therefore, it was assumed that a longitudinal controller was able to maintain a constant longitudinal speed throughout the entire trajectory ($a_{x}=0$). The tests were performed using two different longitudinal velocities: 8 and 12 m/s.

The circuit that was used to generate the path references includes abrupt lateral movements such as a fast double-lane turn and a 180 degree turn. These maneuvers are so aggressive that when driving the real car through this path, the longitudinal velocity was as low as 2.5 m/s in the most critical segments. Therefore, using constant longitudinal velocities of 8 and 12 m/s would allow us to drive close to the vehicle’s dynamic limits.

Moreover, the most realistic tests would assume that new sensor data would be obtained every 0.1 seconds, so M=10. Thus, only in the presence of the DREKF, both controllers would be able to receive new data every T=0.01 s. However, if a single rate EKF (SREKF) were implemented instead of the DREKF, the controllers would have to be calculated every MT=0.1 s.

In this last situation, the MPC-based controller was still able to provide a different control signal every *T* since M≤N, which means that the first *M* discrete-time control signals (M=10) of the control horizon (N=10) would be used at every controller calculation. On the contrary, the IKIBI-based controller had to calculate one control signal every MT in the absence of the DREKF. Finally, both controllers were tested considering process and measurement noises and also without these noises.

### 4.3. Cost Indexes Used to Measure Performance

Two different cost indexes were used in order to better quantify and compare each control solution in each of the tests:

- $J_{1}$, which is based on the $\ell_{2}$-norm, and its goal is to provide a measure of how accurately the path is followed:
  (34) $$J_{1}=\sum_{k=1}^{l}\min_{1\leq k^{\prime }\leq l}\sqrt{{\left(X_{k}-X_{ref,k^{\prime }}\right)}^{2}+{\left(Y_{k}-Y_{ref,k^{\prime }}\right)}^{2}}$$
  where *l* is the number of iterations required by the AGV to reach the final point of the path, ${\left(X,Y\right)}_{k}$ is the current AGV position, and ${\left(X_{ref},Y_{ref}\right)}_{k^{\prime }}$ is the nearest kinematic position reference to the current AGV position.
- $J_{2}$, which is based on the $\ell_{\infty }$-norm and is defined to obtain the maximum difference between the desired path and the current AGV position:
  (35) $$J_{2}=\max_{1\leq k\leq l}\left\{\min_{1\leq k^{\prime }\leq l}\sqrt{{\left(X_{k}-X_{ref,k^{\prime }}\right)}^{2}+{\left(Y_{k}-Y_{ref,k^{\prime }}\right)}^{2}}\right\}$$

## 5. Results and Discussion

This section shows and discusses the results that were obtained from the different tests.

### 5.1. Noiseless, Fast Sensor Feedback Test

The first experiment considered the situation where sensor feedback is received every *T* (fast sampling rate). Therefore, the controllers can also directly calculate the input signal (steering angle) every *T*. Moreover, since it was assumed in this test that there is no measurement or process noises, there was no need for a filter.

**Figure 3:**
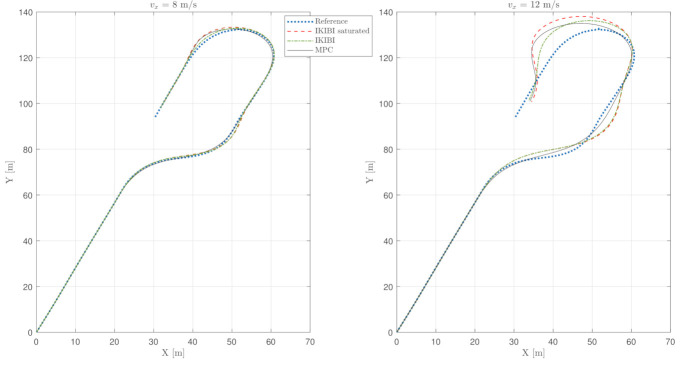
Vehicle path: noiseless, fast sensor feedback test. (**left**) $v_{x}=8$ m/s and (**right**) $v_{x}=12$ m/s.

**Figure 4:**
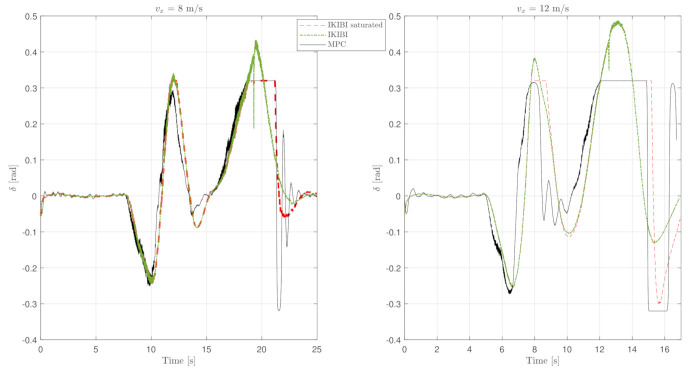
Front wheel steering temporal evolution: noiseless, fast sensor feedback test. (**left**) $v_{x}=8$ m/s and (**right**) $v_{x}=12$ m/s.

**Figure 5:**
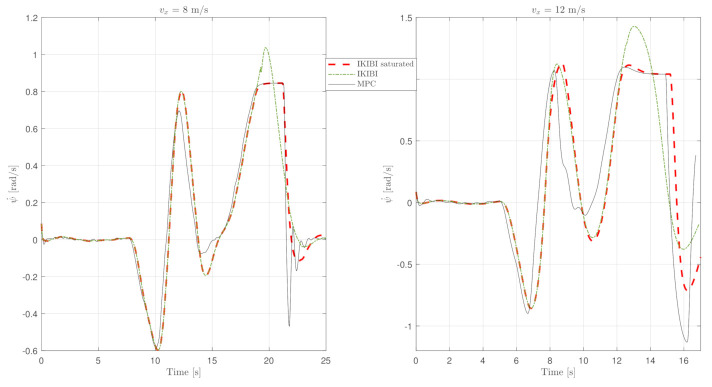
Yaw rate temporal evolution: noiseless, fast sensor feedback test. (**left**) $v_{x}=8$ m/s and (**right**) $v_{x}=12$ m/s.

This test was used to compare each of the two controllers that we proposed for this application. Figure 3, Figure 4 and Figure 5 show the results. Figure 3 plots the X and Y coordinates of each simulation, and Figure 4 and Figure 5 show the temporal evolution of the steering angle and the yaw rate, respectively.

Because of the abruptness of the maneuvers, a degradation of the behavior when the longitudinal velocity of the vehicle is higher is clearly observed in Figure 3. Such an aggressive maneuver is handled by each of the controllers in two different ways.

On the one hand, the IKIBI controller simply increases the steering angle in order to achieve a higher yaw rate. While this may suffice for more moderate maneuvers, in a real scenario, the front wheel angular position is physically bounded, and therefore, the control signal calculated with this controller would not be feasible.

The degradation in the lane-following accuracy when the control signal is saturated for the IKIBI controller with the maximum steering angle of the car can be seen in Figure 3. Here, the front wheels cannot physically turn more than 0.32 radians. This degradation becomes more noticeable the more aggressive the maneuver is; here, the higher the longitudinal velocity is.

On the other hand, MPC can explicitly consider in its calculations that the front wheel steering angle has to be bounded to never violate the physical limitations of the real vehicle. Moreover, because of the prediction horizon, when the car has to perform an abrupt maneuver, the MPC anticipates it and starts steering the wheel before the time that the IKIBI controller does.

As mentioned, Figure 4 shows the front-wheel steering temporal evolution. It can be seen here how the MPC controller is able to keep the steering angle inside the desired boundaries, whereas the IKIBI controller will saturate.

Moreover, Figure 5 plots the temporal evolution of the yaw rate throughout the trajectory. As mentioned earlier, because of the predictive nature of the MPC controller in a longer term horizon than the IKIBI controller, it is able to anticipate when a big turn is required and starts steering the vehicle earlier than the other controller analyzed.

As a consequence, the trajectory whose input references were generated by MPC will be smoother. Moreover, MPC can explicitly control the feeling of comfort experienced by the vehicle passengers using the expression (14). Since this expression acts by limiting the yaw rate, the driving experience will be more satisfying when using MPC rather than the IKIBI controller.

Finally, Table 1 shows the performance cost indexes for each of the controllers in this fast, noiseless test. It can be seen how, by explicitly considering the physical limitations of the vehicle such as the maximum front-wheel steering angle over a prediction horizon, the MPC is able to follow the reference path more accurately than its IKIBI counterpart.

### 5.2. Fast Sensor Feedback Test with Noise Using EKF

Process and measurement noises are present in a real scenario for this lane-keeping application. Unfortunately, the previous test was observed to turn unstably if these noises were present. Thus, the use of EKF is justified.

**Figure 6:**
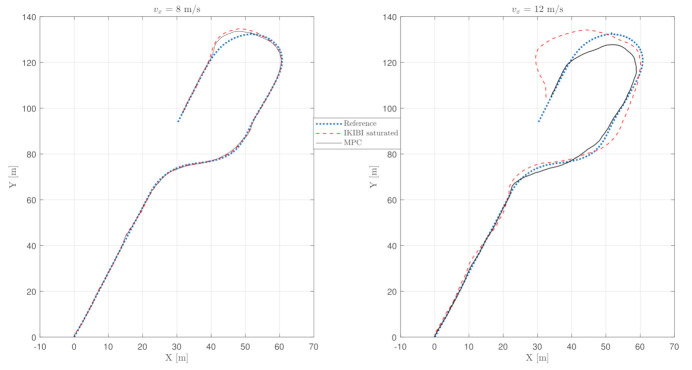
Vehicle path: fast sensor feedback test with noise using EKF. (**left**) $v_{x}=8$ m/s and (**right**) $v_{x}=12$ m/s.

Figure 6 plots the planar coordinates of the trajectories in the case where both of these noises are present and an EKF is implemented. As mentioned, since using an EKF is essential to have a stable trajectory, we will not show the unstable results for the tests that did not consider using the EKF.

The behavior seen in Figure 6 is analogous to the former experiment that did not consider noises: the behavior degrades when increasing the longitudinal velocity of the vehicle, and the IKIBI controller is saturated. On the other hand, MPC is still able to control the system from this velocity.

Table 2 shows a quantitative version of what is graphically presented in Figure 6. The MPC controller allows a more accurate lane-keeping behavior when compared to the proposed IKIBI controller, and this is accentuated the more extreme the situation is: in the presence of measurement and process noises and with high longitudinal velocities.

### 5.3. Noiseless, Slow Sensor Feedback Test

Nonetheless, the most relevant situation occurs when sensor measurements are not updated every *T*, but they are updated every MT (here, M=10). In this situation, the controllers have to be calculated *M*-times slower than in the previous situations. This test explores the situation where no EKF is used and there is no measurement or process noise.

For the IKIBI controller, this situation will necessarily involve keeping the control action constant throughout MT. However, MPC is capable of acting differently. Even though usually, MPC calculates a control sequence over a whole prediction horizon, but only the first control action of this sequence is applied, it is also possible to apply the different control actions of the control horizon if the update rate of the MPC calculations is not fast enough.

**Figure 7:**
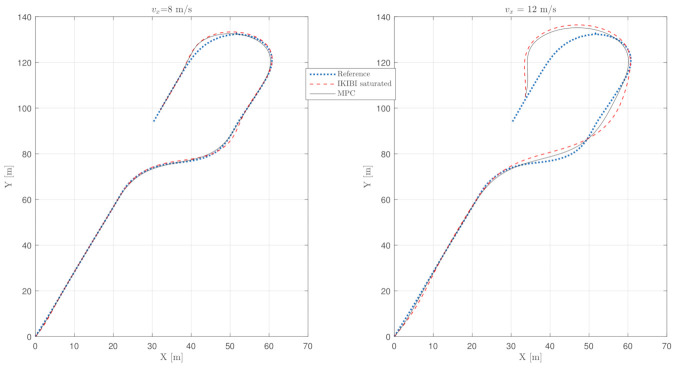
Vehicle path test: noiseless, slow sensor feedback test: (**left**) $v_{x}=8$ m/s and (**right**) $v_{x}=12$ m/s.

Figure 7 shows the comparison between an IKIBI controller calculated every MT and an MPC controller that is calculated every MT, but updates its control signal every *T* because it uses its entire control horizon.

The disadvantage of this implementation strategy for the MPC controller is that the anticipation ability of MPC is lost, especially in this application where the control horizon is equal to the prediction horizon. As a consequence, the lane-keeping behavior degrades, as seen in Table 3.

However, the MPC strategy is still a more accurate option than the IKIBI controller because of its ability to explicitly constrain physical variables such as the steering angle of the front wheels.

### 5.4. Slow Sensor Feedback Test with Noise Using the DREKF

Finally, we also considered the situation where the sensor feedback was obtained at a slow rate (every MT) and there were process and measurement noises. The initial test performed in these conditions was to analyze the behavior of each of the two controllers when a Single-Rate EKF (SREKF) was used with a slow sampling frequency. The controllers were also meant to be calculated every MT. However, neither of the two controller strategies (MPC and IKIBI) were able to produce a stable lane-keeping behavior in this situation.

**Figure 8:**
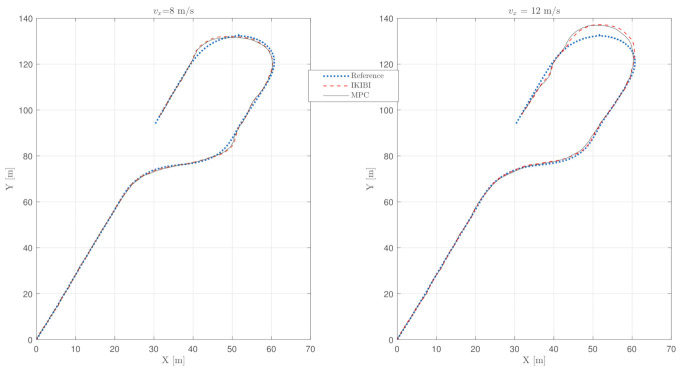
Vehicle path: slow sensor feedback test with noise using the DREKF. (**left**) $v_{x}=8$ m/s (**right**) $v_{x}=12$ m/s.

Thus, the use of a Dual-Rate EKF (DREKF) is necessary. The DREKF has the ability to provide new measurements every *T* while only updating its internal matrices and acquires measured variables every MT. Figure 8 shows the results for implementing the DREKF to calculate both controllers every *T* while only receiving new sensor data every MT. Moreover, Table 4 shows the cost indexes for this experiment. It can be seen how the DREKF allows an accurate lane-keeping behavior in situations where only slow and noisy sensor feedback is available.

## 6. Conclusions

The formulation of the model predictive control problem is especially well suited for controlling self-driving cars since it is able to take into consideration long prediction horizons that would be especially important in the event of abrupt maneuvers and in the presence of measurement and process noise.

Additionally, the physical limitations of the vehicle can be explicitly considered, and the comfort of the passengers can be directly taken into consideration by using this control scheme.

For these reasons, MPC provides a more accurate lane-keeping behavior than an IKIBI control strategy. The difference in the accuracy of each of the two controllers can be quantified by the cost indexes introduced in Section 4.

The use of EKF has been essential to obtain a stable behavior of the system in this application when measurement and process noises are present. If the update rate of the sensor data is fast enough, it will suffice to use a standard EKF, called the SREKF in this work.

However, if the update rate of the filter’s internal state is too slow, a DREKF should be used, since it will allow providing new variable estimations every *T* to the controllers so that they can be calculated at a fast rate while updating the internal state every MT.

One alternative to the use of a DREKF would be to use all the input sequence of the control horizon when calculating the MPC controller every MT. Nonetheless, this is a suboptimal solution since there is a loss of the anticipation ability, which is characteristic of MPC. Furthermore, this alternative is only feasible when noise is not present, which happens scarcely in a real application.

Finally, we observe that including a DREKF allows obtaining a degree of lane-keeping accuracy with a slow and noisy sensor feedback similar to the one obtained for the test where there was no noise and sensor data were acquired at a fast rate for both proposed controllers.

## Figures and Tables

**Table 1:** Cost indexes: noiseless, fast sensor feedback test.

Controller	$v_{x}=8$ m/s		$v_{x}=12$ m/s	
	$J_{1}$ (m)	$J_{2}$ (m)	$J_{1}$ (m)	$J_{2}$ (m)
IKIBI	492.13	0.9	2090.2	5.15
IKIBI saturated	667.3	1.88	3036.1	8.39
MPC	561	1.67	1817.2	6.5

**Table 2:** Cost indexes: fast sensor feedback test with noise using EKF.

Controller	$v_{x}=8$ m/s		$v_{x}=12$ m/s	
	$J_{1}$ (m)	$J_{2}$ (m)	$J_{1}$ (m)	$J_{2}$ (m)
IKIBI saturated	999.4	3.42	3660.9	10.77
MPC	834.3	2.63	1269.5	4.54

**Table 3:** Cost indexes: noiseless, slow sensor feedback test.

Controller	$v_{x}=8$ m/s		$v_{x}=12$ m/s	
	$J_{1}$ (m)	$J_{2}$ (m)	$J_{1}$ (m)	$J_{2}$ (m)
IKIBI saturated	800.9	1.91	3039.8	7.49
MPC	613.8	1.69	2026.6	6.86

**Table 4:** Cost indexes: slow sensor feedback test with noise using the DREKF.

Controller	$v_{x}=8$ m/s		$v_{x}=12$ m/s	
	$J_{1}$ (m)	$J_{2}$ (m)	$J_{1}$ (m)	$J_{2}$ (m)
IKIBI saturated	764.76	1.58	1057.3	4.67
MPC	738	1.3	1040.2	4.75

## Data Availability

Not Applicable.

## Abbreviations

The following abbreviations are used in this manuscript:

MPC	Model Predictive Control
IKIBI	Inverse Kinematics Bicycle
EKF	Extended Kalman Filter
AGV	Autonomous Ground Vehicle
LPV	Linear Parameter Varying
IMU	Inertial Measurement Unit
DOF	Degrees of Freedom

## Appendix A. Simulation Model

Simulations were performed using MATLAB Simulink’s Vehicle Dynamics Blockset. This toolbox is equipped with a Vehicle Body 3DOF block that implements a rigid two-axle vehicle body model to calculate longitudinal, lateral, and yaw motion. The dynamic equations of the internal model used in this block are [39]:

(A1) $$\begin{array}{ccc} \ddot{y} & = & -\dot{x}r+\frac{F_{yf}+F_{yr}+F_{y,\mathrm{ext}}}{m} \end{array}$$

(A2) $$\begin{array}{ccc} \dot{r} & = & \frac{aF_{yf}-bF_{yr}+M_{z,\mathrm{ext}}}{I_{zz}} \end{array}$$

(A3) $$\begin{array}{cc} \dot{\Psi } & =r \end{array}$$

where $F_{yf}$ and $F_{yr}$ are the lateral forces applied to the front and rear wheels, respectively, along the vehicle-fixed y-axis. Additionally, the external forces that act on the vehicle center of gravity are:

(A4) $$\begin{array}{ccc} F_{x/y/z,\mathrm{ext}} & = & F_{d,x/y/z}+F_{x/y/z,\mathrm{input}} \end{array}$$

(A5) $$\begin{array}{ccc} M_{x/y/z,\mathrm{ext}} & = & M_{d,x/y/z}+M_{x/y/z,\mathrm{input}} \end{array}$$

where the subindex ${}_{d}$ indicates that they are drag forces/moments. Then:

(A6) $$\begin{array}{ccc} F_{xft} & = & 0 \end{array}$$

(A7) $$\begin{array}{ccc} F_{xft} & = & -C_{\alpha f}\alpha_{f}\mu_{f}\frac{F_{zf}}{F_{z,\mathrm{nom}}} \end{array}$$

(A8) $$\begin{array}{ccc} F_{xrt} & = & 0 \end{array}$$

(A9) $$\begin{array}{ccc} F_{xrt} & = & -C_{\alpha r}\alpha_{r}\mu_{r}\frac{F_{zr}}{F_{z,\mathrm{nom}}} \end{array}$$

where the subindex ${}_{t}$ indicates that the forces are acting on the tires. Here,

(A10) $$\begin{array}{c} F_{zf}=\frac{bmg-\left(\ddot{x}-\dot{y}r\right)mh+hF_{x,\mathrm{ext}}+bF_{z,\mathrm{ext}}-M_{y,\mathrm{ext}}}{a+b} \end{array}$$

(A11) $$\begin{array}{c} F_{zr}=\frac{amg+\left(\ddot{x}-\dot{y}r\right)mh-hF_{x,\mathrm{ext}}+aF_{z,\mathrm{ext}}+M_{y,\mathrm{ext}}}{a+b} \end{array}$$

Moreover, the tire forces can be calculated with the slip angles (α):

(A12) $$\begin{array}{ccc} \alpha_{f} & = & \arctan\left(\frac{\dot{y}+ar}{\dot{x}}\right)-\delta_{f} \end{array}$$

(A13) $$\begin{array}{ccc} \alpha_{r} & = & \arctan\left(\frac{\dot{y}-br}{\dot{x}}\right)-\delta_{r} \end{array}$$

(A14) $$\begin{array}{ccc} F_{xf} & = & F_{xft}\cos\left(\delta_{f}\right)-F_{yft}\sin\left(\delta_{f}\right) \end{array}$$

(A15) $$\begin{array}{ccc} F_{yf} & = & -F_{xft}\sin\left(\delta_{f}\right)+F_{yft}\cos\left(\delta_{f}\right) \end{array}$$

(A16) $$\begin{array}{ccc} F_{xr} & = & F_{xrt}\cos\left(\delta_{r}\right)-F_{yrt}\sin\left(\delta_{r}\right) \end{array}$$

(A17) $$\begin{array}{ccc} F_{yr} & = & -F_{xrt}\sin\left(\delta_{r}\right)+F_{yrt}\cos\left(\delta_{r}\right) \end{array}$$

The physical variables needed to calculate these equations are:

- *m*, the vehicle body mass.
- *a* and *b*, the distance of the front and rear wheels, respectively, from the normal projection point of vehicle’s CG onto the common axle plane.
- $I_{zz}$, the vehicle body moment of inertia about the vehicle-fixed z-axis.
- $C_{\alpha }$, cornering stiffness. This constant represents a linear approximation for the relationship between the slip angle, α, and the lateral force, $F_{y}$.
- μ, the wheel friction coefficient.
- *h*, the height of the vehicle’s center of gravity above the axle plane.

Furthermore, subscripts ${}_{f}$ and ${}_{r}$ refer to the front and rear axles, respectively.

Finally, Table A1 shows the numerical values used for the constants presented earlier.

**Table A1:** Simulation model constants.

Constant	Value	Unit
m	1800	kg
a	1.6	m
b	1.65	m
$\mu_{f}$	0.6	-
$\mu_{r}$	0.6	-
$C_{\alpha ,f}$	120	kN/rad
$C_{\alpha ,r}$	110	kN/rad
h	0.35	m
$I_{zz}$	3270	$\mathrm{kg}\cdot m^{2}$
